# Co-Therapy of Albendazole and Dexamethasone Reduces Pathological Changes in the Cerebral Parenchyma of Th-1 and Th-2 Dominant Mice Heavily Infected with *Angiostrongylus cantonensis*: Histopathological and RNA-seq Analyses

**DOI:** 10.3390/biom11040536

**Published:** 2021-04-06

**Authors:** Kai-Yuan Jhan, Chien-Ju Cheng, Shih-Ming Jung, Yi-Jen Lai, Kuang-Yao Chen, Lian-Chen Wang

**Affiliations:** 1 Graduate Institute of Biomedical Sciences, College of Medicine, Chang Gung University, Taoyuan 333, Taiwan; ed790101@hotmail.com (K.-Y.J.); ericlai820103@gmail.com (Y.-J.L.); 2 Department of Parasitology, College of Medicine, Chang Gung University, Taoyuan 333, Taiwan; d000014521@cgu.edu.tw; 3 Department of Pathology, Chang-Gung Memorial Hospital, Chang-Gung Children Hospital at Linkou and Chang-Gung University, Taoyuan 333, Taiwan; ming22@cgmh.org.tw; 4 Department of Parasitology, School of Medicine, China Medical University, Taichung 404, Taiwan; 5 Molecular Infectious Disease Research Center, Chang Gung Memorial Hospital, Taoyuan 333, Taiwan

**Keywords:** *Angiostrongylus cantonensis*, albendazole, dexamethasone, histopathology, pathological changes, RNA-seq, scoring

## Abstract

Administration of albendazole alone was not very suitable for the treatment of cerebral angiostrongyliasis. This study was designed to evaluate the effects of the co-therapy of this drug and dexamethasone in Th-1 and Th-2 dominant mice infected with *Angiostrongylus cantonensis*. Each of BALB/c and C57BL/6 mice infected with 50 *A. cantonensis* third-stage larvae were administered albendazole (10 mg/kg/day) alone, dexamethasone (0.5 mg/kg/day) alone, or co-therapy of the two drugs from day 7 or 14 post-infection for 7 or 14 days. After sacrifice, coronal slices were prepared from five brain regions and stained with hematoxylin and eosin. Eight pathological changes were employed to determine the therapeutic effectiveness using a scoring system. RNA-seq analysis was performed to confirm the histopathological findings. The infected BALB/c and C57BL/6 mice had similar patterns in the pathological changes. Meningitis, hemorrhage, size of worms, and encephalitis in the cerebral parenchyma were slighter in the mice treated with co-therapy than the remaining groups. Mice treated from day 14 had more severe changes than those from day 7. The histopathological findings were found to be consistent to immune responses determined by RNA-seq analysis. Co-therapy was determined to reduce pathological changes after administration to mice infected with *A. cantonensis*.

## 1. Introduction

In the recent decade, cerebral angiostrongyliasis caused by *Angiostrongylus cantonensis* has become an emerging disease in many parts of the world [1,2,3]. The clinical manifestations of this infection are associated with its intensity. Light infections may lead to headache, fever, shoulder and neck pain, sensitive and stinging of the skin, visual impairments, and other neglectable symptoms. These slight manifestations may be overlooked and recovered spontaneously. In heavy infections, severe headaches, fever, nausea, vomiting, neck stiffness, and neurologic abnormalities may persist for weeks to months. Moreover, larval migration in the brain may also cause permanent mechanical damages [4].

It is still controversial on whether administer anthelmintics and/or corticosteroids for the treatment of cerebral angiostrongyliasis. Albendazole has been reported to be effective against *A. cantonensis* infection in mice treated within 15 days post-infection [5,6]. However, this drug causes more severe pathological changes in the brains of infected rabbits than their untreated counterparts, suggesting that albendazole may not be very suitable for the treatment of cerebral angiostrongyliasis [7]. To overcome these adverse effects, co-therapy of anthelmintics and corticosteroids has been deemed to be beneficial in the treatment of *A*. *cantonensis* infections in mice [8,9,10]. Moreover, dexamethasone has been documented to inhibit apoptosis in mice with *A*. *cantonensis* [11]. A one-week co-therapy of albendazole and dexamethasone appears to be safe and beneficial to the treatment of angiostrongyliasis [12,13].

Although the effectiveness of co-therapy of albendazole and dexamethasone on the treatment of cerebral angiostrongyliasis has been reports [10,12,13], these reports were mainly based on biochemical or clinical results. Observations on the effects of the co-therapy on pathological changes in the cerebral parenchyma of infected individuals may not complete. In our previous study, the brain was divided into different regions and examined by histopathological and immunohistochemical techniques. The results demonstrated the temporal-spatial pathological changes in the brains of mice infected with *A. cantonensis* [14]. Moreover, in our previous immunohistochemical study on IL-4, IL-10, and IL-13 in the brains of infected BALB/c and C57BL/6 mice, a special temporal-spatial expression was revealed in the cerebral parenchyma and provided explanation the differences in the survival and the time of occurrence of immune responses between the infected hosts [15]. These two murine strains are inbred lines with different H-2 complex and the major histocompatibility complex (MHC) of the former is H-2^b^ whereas the latter H-2^d^. They have significant differences in pathogen tolerance and the severity of symptoms or immune activation [16,17]. Comparison of these strains may be useful to evaluate the pathological changes after different chemotherapeutic strategies and to select the suitable animal model for further investigations.

RNA-seq is a recent approach using the next-generation sequencing technology to study changes in the entire transcriptome [18]. This technique has not only employed to study bacterial and viral pathogens [19,20] but also in parasitology research [21,22,23,24,25]. In addition to histopathological examination of lesions in the cerebral parenchyma, we also employed RNA-seq to confirm the effects of albendazole alone, dexamethasone alone, and co-therapy of the two drugs. The co-therapy strategy was determined to be effective in reducing pathological changes in the cerebral parenchyma of the heavily infected Th-1 and Th-2 dominant mice.

## 2. Materials and Methods

### 2.1. Parasite and Laboratory Animals

A Taiwan strain of *A. cantonensis* was employed in this study. Its life cycle has been maintained in our laboratory through *Biomphalaria glabrata* snails and Sprague-Dawley (SD) rats since 1985 [15]. BALB/c (H-2^d^) mice (8 weeks old) and SD rats for life-cycle maintenance were purchased from the National Laboratory Animal Center (Taipei, Taiwan). C57BL/6 (H-2^b^) mice (8 weeks old) were purchased from BioLASCO Taiwan Co., Ltd. (Taipei, Taiwan). All procedures involving the experimental animals and their care were reviewed and approved by the Chang Gung University Institutional Animal Care and Use Committee (IACUC).

### 2.2. Experimental Infection

L3 of *A. cantonensis* were isolated from infected snails by digesting with 0.6% (*w*/*v*) pepsin-HCl (pH 2-3) for 1 h [26]. Each mouse was inoculated with 50 larvae by stomach intubation. The infected animals were separately housed in plastic cages and provided with food and drinking water ad libitum.

### 2.3. Drug Administration

From day 7 or 14 post-infection, each mouse received albendazole (Sigma-Aldrich, St. Louis, MO, USA) (10 mg/kg/day) alone, dexamethasone (Taiwan Biotech Group, Taoyuan, Taiwan) (0.5 mg/kg/day) alone, or co-therapy of the two drugs for 7 or 14 days. Albendazole was administered by stomach intubation and dexamethasone by intraperitoneal injection.

### 2.4. Histopathological Examination

The mice were sacrificed by inhalation of 3% (*v*/*v*) isoflurane (Panion & BF Biotech Inc., Taipei, Taiwan). Brains were removed from the cranial cavity and fixed in 10% formalin. After fixation for three weeks, the brains were embedded in paraffin. Each brain was cut into five sections. From each section, coronal slices (2–6 µm) were prepared and then stained with haematoxylin and eosin [14].

Regions of the mouse brain were divided according to the Allen Brain Atlas [27]. Each brain was divided into five sections including the anterior cerebrum, lateral ventricles, third ventricle and hippocampus, posterior cerebrum and fourth ventricle, and cerebellum. The stained slices prepared from each section were examined under a light microscope.

Eight pathological changes were employed to determine the effectiveness of the therapeutic strategies. These changes included eosinophilic meningitis, brain necrosis, choriomeningitis, size/finding of larvae, meninges congestion, perivascular cuffing, encephalitis, and hemorrhage. After determining the area of each change in the slices, medians were calculated and employed as a cut-off point for severity. Negative results were scored 0, scores of 1 represented those below or equal to the median, and scores of 2 indicated those above the median. A score of severity for each pathological change was calculated by strain of mice (BALB/c and C57BL/6 mice), chemotherapeutic strategies (albendazole, dexamethasone, and co-therapy), starting time of drug administration (day 7 and day 14), and duration of administration (7 days and 14 days).

### 2.5. RNA-seq

After removal from the cranial cavity, total RNA was extracted from the brains using the GENEzol TriRNA Pure Kit (Geneaid, New Taipei, Taiwan). The concentration of RNA was determined with a spectrophotometer (OD260 nm). In addition, RNA purity and quantification were checked using SimpliNano™—Biochrom Spectrophotometers (Biochrom, MA, USA) and its degradation and integrity were monitored by Qsep 100 DNA/RNA Analyzer (BiOptic Inc., Taiwan). From each sample, 1 µl of total RNA was used as an input material for RNA sample preparations. Sequencing libraries were generated using KAPA mRNA HyperPrep Kit (KAPA Biosystems, Roche, Basel, Switzerland) according to the instructions of the manufacturer and index codes were added to the attribute sequences of each sample. Differentially expressed genes (DEGs) analysis of the three chemotherapeutic strategies was performed in R using DEGseq and DEGseq2 based on negative binominal distribution and Poisson distribution model, respectively [28,29,30].

### 2.6. Statistical Analysis

Data are expressed as mean ± standard error of the mean. Differences among the groups are analyzed by ANOVA and *p* < 0.05 was considered to be statistically significant.

## 3. Results

### 3.1. Pathological Changes

**Figure 1:**
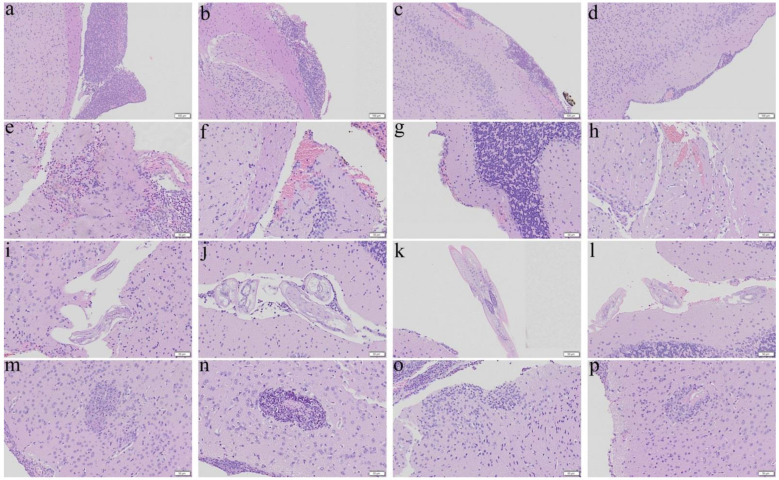
Representative photomicrographs showing meningitis, presence of worms, hemorrhage, and encephalitis in the cerebral parenchyma of the BALB/c mice infected with *Angiostrongylus cantonensis* treated from day 7 for 7 days. (**a**,**e**,**i**,**m**) Untreated mice. (**b**,**f**,**j**,**n**) Mice treated with albendazole alone. (**c**,**g**,**k**,**o**) Mice treated with dexamethasone alone. (**d**,**h**,**l**,**p**) Co-therapy of albendazole and dexamethasone.

**Figure 2:**
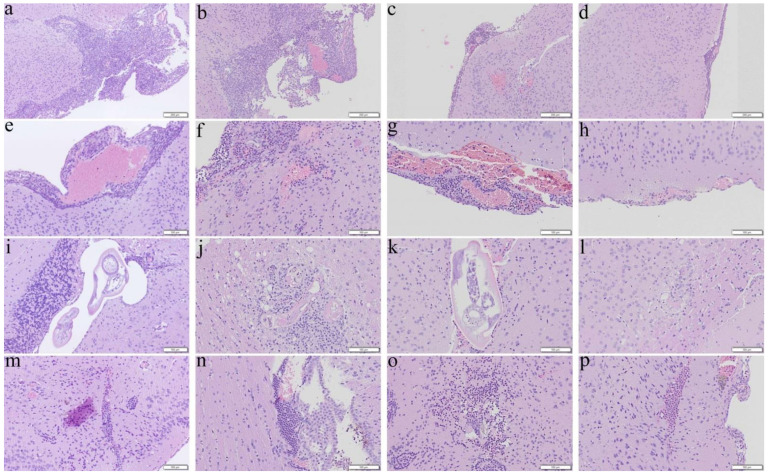
Representative photomicrographs showing meningitis, presence of worms, hemorrhage, and encephalitis in the cerebral parenchyma of the BALB/c mice infected with *Angiostrongylus cantonensis* treated from day 7 for 14 days. (**a**,**e**,**i**,**m**) Untreated mice. (**b**,**f**,**j**,**n**) Mice treated with albendazole alone. (**c**,**g**,**k**,**o**) Mice treated with dexamethasone alone. (**d**,**h**,**l**,**p**) Co-therapy of albendazole and dexamethasone.

**Figure 3:**
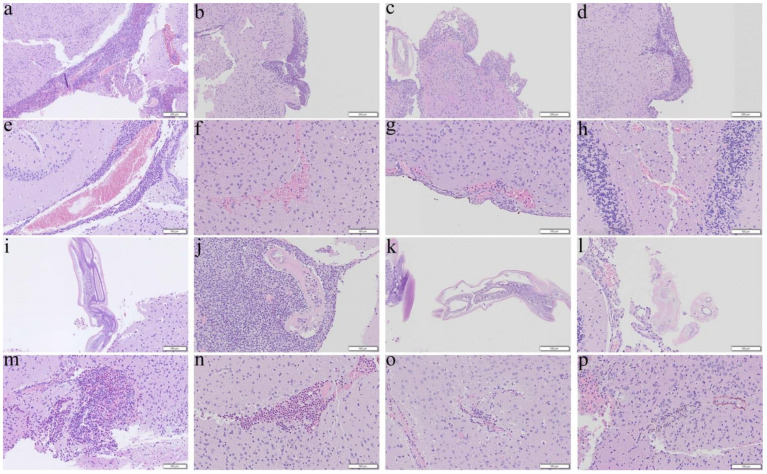
Representative photomicrographs showing meningitis, presence of worms, hemorrhage, and encephalitis in the cerebral parenchyma of the BALB/c mice infected with *Angiostrongylus cantonensis* treated from day 14 for 7 days. (**a**,**e**,**i**,**m**) Untreated mice. (**b**,**f**,**j**,**n**) Mice treated with albendazole alone. (**c**,**g**,**k**,**o**) Mice treated with dexamethasone alone. (**d**,**h**,**l**,**p**) Co-therapy of albendazole and dexamethasone.

**Figure 4:**
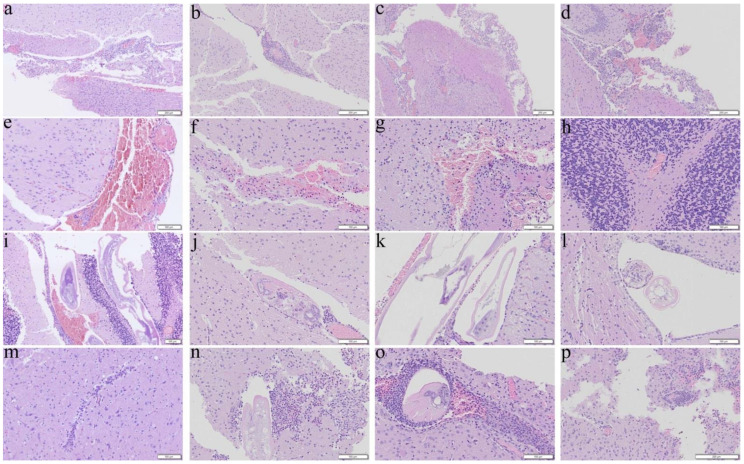
Representative photomicrographs showing meningitis, presence of worms, hemorrhage, and encephalitis in the cerebral parenchyma of the BALB/c mice infected with *Angiostrongylus cantonensis* treated from day 14 for 14 days. (**a**,**e**,**i**,**m**) Untreated mice. (**b**,**f**,**j**,**n**) Mice treated with albendazole alone. (**c**,**g**,**k**,**o**) Mice treated with dexamethasone alone. (**d**,**h**,**l**,**p**) Co-therapy of albendazole and dexamethasone.

Figure 1, Figure 2, Figure 3 and Figure 4 show meningitis, hemorrhage, finding of worms, and encephalitis in the cerebral parenchyma of the untreated BALB/c mice and those treated with albendazole alone, dexamethasone alone, and co-therapy of the two drugs. Meningitis and encephalitis became slight in the co-therapy group treated from day 7 for 7 days (Figure 1d,p) whereas these changes remained very severe in the albendazole (Figure 1b,n) and dexamethasone (Figure 1c,o) alone groups. However, hemorrhage (Figure 1f–h) and finding of worms (Figure 1j–l) were found in the three groups of treated mice.

Mice received the co-therapy from day 7 for 14 days had improvements in reducing the four changes (Figure 2d,h,l,p) whereas meningitis (Figure 2b) and encephalitis (Figure 2n) remained severe in the albendazole group, although hemorrhage (Figure 2f) and finding of worms (Figure 2j) became unapparent.

The co-therapy (Figure 3d,p) and dexamethasone (Figure 3c,o) groups administered from day 14 for 7 days were also determined to have reduction in meningitis and encephalitis whereas no changes in severities were found in mice treated with albendazole alone (Figure 3b,n). In addition, hemorrhage (Figure 3f–h) and finding of worms (Figure 3j–l) were also observed in the treated mice.

Reduction in severities of meningitis and encephalitis were found in the dexamethasone (Figure 4c,o) and co-therapy (Figure 4d,p) groups treated from day 14 for 14 days. However, only reduction in hemorrhage and finding of worms was found in the co-therapy group (Figure 4h,l).

**Figure 5:**
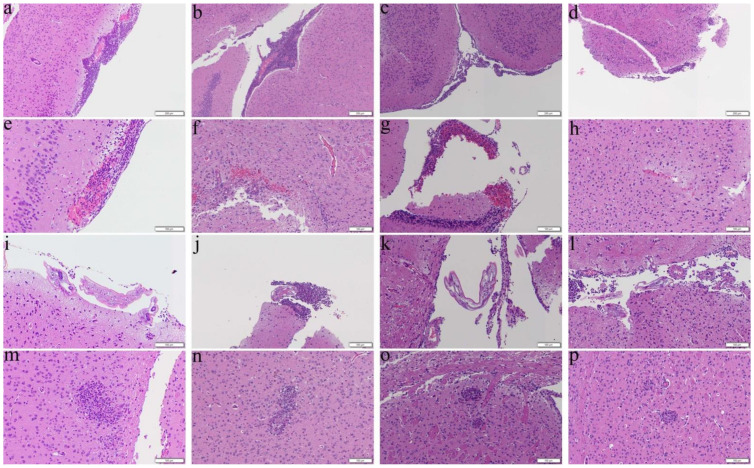
Representative photomicrographs showing meningitis, presence of worms, hemorrhage, and encephalitis in the cerebral parenchyma of the C57BL/6 mice infected with *Angiostrongylus cantonensis* from day 7 for 7 days. (**a**,**e**,**i**,**m**) Untreated mice. (**b**,**f**,**j**,**n**) Mice treated with albendazole alone. (**c**,**g**,**k**,**o**) Mice treated with dexamethasone alone. (**d**,**h**,**l**,**p**) Co-therapy of albendazole and dexamethasone.

Similarly to BALB/c mice treated, C57BL/6 in the co-therapy group from day 7 for 7 days had reduction in meningitis and encephalitis (Figure 5d,p) while no improvements were observed in the remaining two groups (Figure 5b,c,n,o). Hemorrhage (Figure 5f–h) and finding of worms (Figure 5j–l) were revealed in the three groups of treated mice.

**Figure 6:**
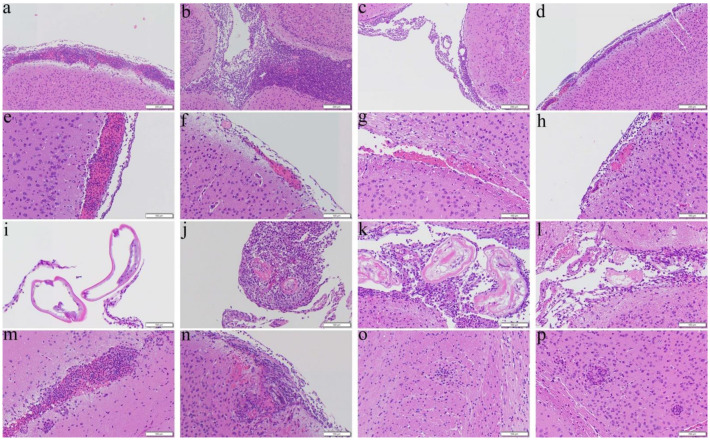
Representative photomicrographs showing meningitis, presence of worms, hemorrhage, and encephalitis in the cerebral parenchyma of the C57BL/6 mice infected with *Angiostrongylus cantonensis* treated from day 7 for 14 days. (**a**,**e**,**i**,**m**) Untreated mice. (**b**,**f**,**j**,**n**) Mice treated with albendazole alone. (**c**,**g**,**k**,**o**) Mice treated with dexamethasone alone. (**d**,**h**,**l**,**p**) Co-therapy of albendazole and dexamethasone.

Mice in the dexamethasone (Figure 6c,o) and co-therapy (Figure 6d,p) groups from day 7 from 14 days also had improvements in meningitis and encephalitis. However, reduction in hemorrhage (Figure 6f) and finding of worms and (Figure 6j) occurred in the albendazole group.

**Figure 7:**
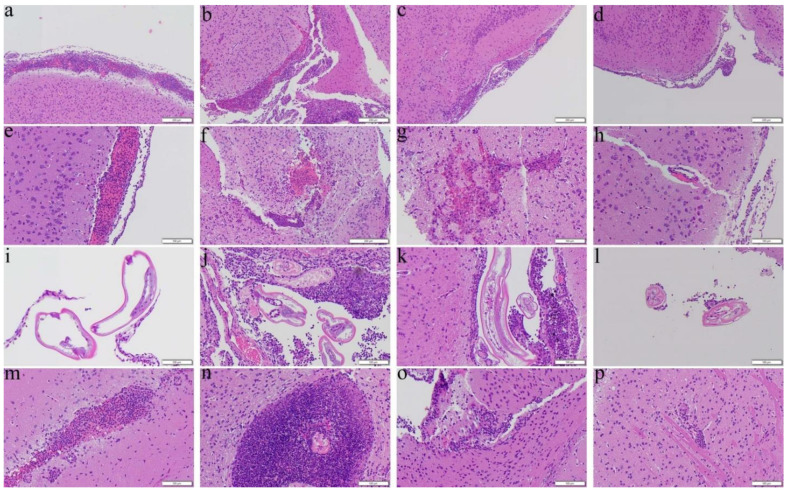
Representative photomicrographs showing meningitis, presence of worms, hemorrhage, and encephalitis in the cerebral parenchyma of the C57BL/6 mice infected with *Angiostrongylus cantonensis* treated from day 14 for 7 days. (**a**,**e**,**i**,**m**) Untreated mice. (**b**,**f**,**j**,**n**) Mice treated with albendazole alone. (**c**,**g**,**k**,**o**) Mice treated with dexamethasone alone. (**d**,**h**,**l**,**p**) Co-therapy of albendazole and dexamethasone.

**Figure 8:**
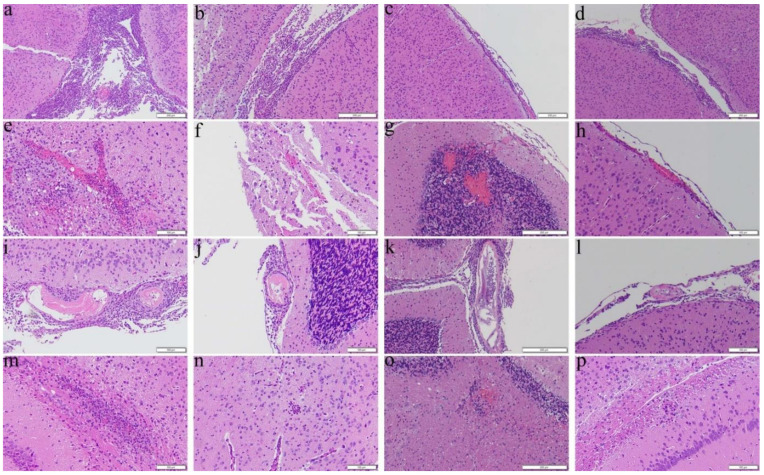
Representative photomicrographs showing meningitis, size of worms, hemorrhage, and encephalitis in the cerebral parenchyma of the C57BL/6 mice infected with *Angiostrongylus cantonensis* treated from day 14 for 14 days. (**a**,**e**,**i**,**m**) Untreated mice. (**b**,**f**,**j**,**n**) Mice treated with albendazole alone. (**c**,**g**,**k**,**o**) Mice treated with dexamethasone alone. (**d**,**h**,**l**,**p**) Co-therapy of albendazole and dexamethasone.

Mice in the co-therapy (Figure 7d,p) and dexamethasone (Figure 7c,o) groups from day 14 for 7 days had reduction in meningitis and encephalitis while no changes in severities were found in the albendazole group (Figure 7b,h). In addition, hemorrhage (Figure 7f–h) and finding of worms (Figure 7j–l) were also found in the three groups of treated mice. Although severities of meningitis and encephalitis were reduced in the dexamethasone (Figure 8c,o) and co-therapy (Figure 8d,p) groups treated from day 14 for 14 days, hemorrhage and finding of worms became unapparent only in the co-therapy group (Figure 8h,l).

### 3.2. Scoring

**Figure 9:**
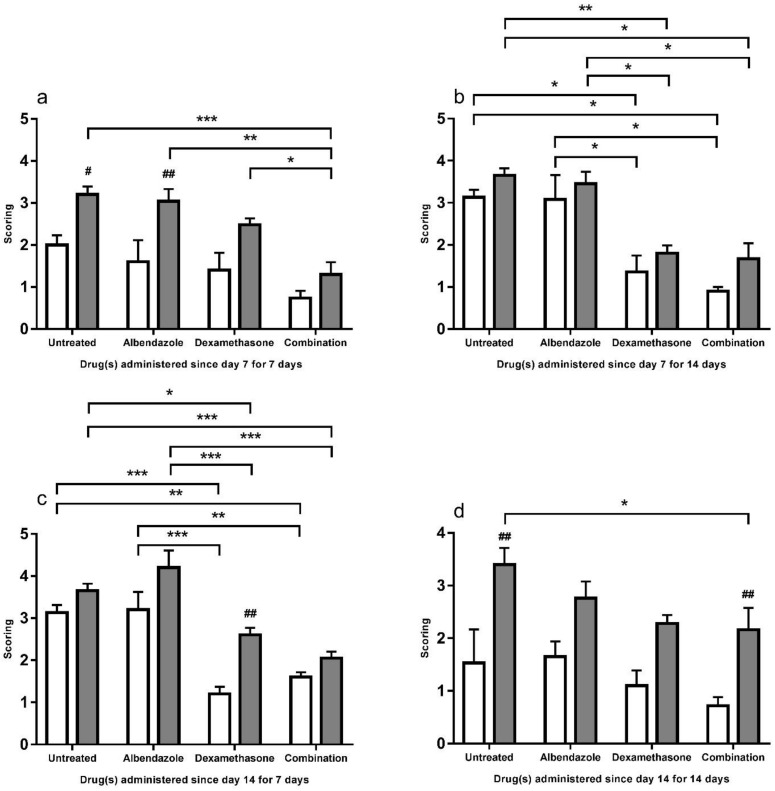
Scorings of brain necrosis and choriomeningitis in the cerebral parenchyma of the BALB/c (white) and C57BL/6 (gray) untreated mice and those treated with albendazole alone dexamethasone alone, and co-therapy of the two drugs after infection with *Angiostrongylus cantonensis*. Data are expressed as mean ± standard error of the mean (untreated group vs. albendazole group vs. dexamethasone group vs. co-therapy group, * *p* < 0.05, ** *p* < 0.01, *** *p* < 0.001) (BALB/c mice vs. C57BL/6 mice, # *p* < 0.05, ## *p* < 0.01)).

The albendazole groups of the C57BL/6 mice had a significant higher scoring in brain necrosis and choriomeningitis than the remaining two groups (*p* < 0.01) from day 7 for 7 days (Figure 9a). Significantly higher scorings were also found in this murine strain treated with dexamethasone (*p* < 0.01) alone from day 14 for 7 days (Figure 9c) and combination of two drugs (*p* < 0.01) from day 14 for 14 days (Figure 9d). The C57BL/6 mice received co-therapy had significantly lower scorings than those treated with dexamethasone (*p* < 0.05) or albendazole (*p* < 0.01) alone from day 7 for 7 days (Figure 9a). From day 7 for 14 days (Figure 9b), the C57BL/6 mice treated with co-therapy and dexamethasone groups had significantly lower scorings than those with albendazole alone (*p* < 0.05). Among C57BL/6 mice treated from day 14 for 7 days (Figure 9c), the co-therapy (*p* < 0.01) or dexamethasone group (*p* < 0.001) had significantly lower scorings than the albendazole group. C57BL/6 mice received co-therapy from day 14 for 7 days (Figure 9c) had significantly lower scoring than those without treatment (*p* < 0.05). In BALB/c mice, the co-therapy (*p* < 0.05) or dexamethasone (*p* < 0.05) group had significantly lower scorings than the albendazole group in those treated from day 7 for 14 days (Figure 9b) or from day 14 for 7 days (Figure 9c).

**Figure 10:**
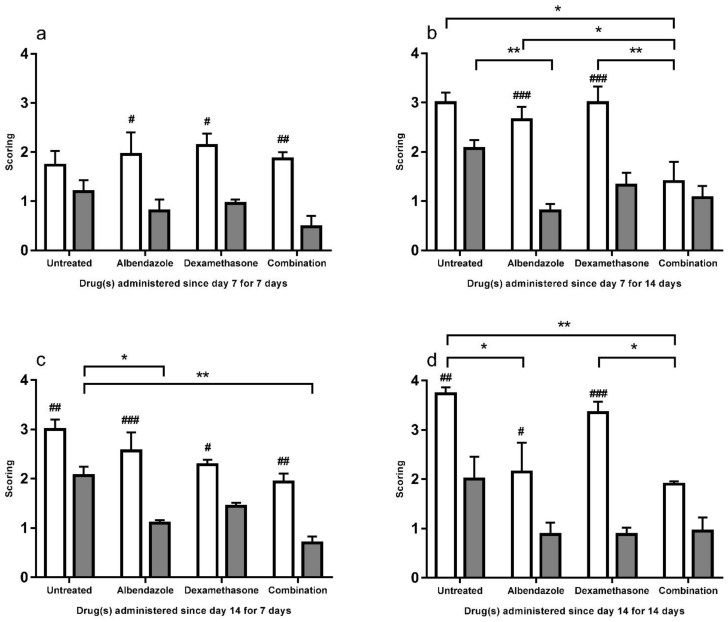
Scorings of meninges congestion and hemorrhage in the cerebral parenchyma of the BALB/c (white) and C57BL/6 (gray) untreated mice and those treated with albendazole alone dexamethasone alone, and co-therapy of the two drugs after infection with *Angiostrongylus cantonensis*. Data are expressed as mean ± standard error of the mean (Untreated group vs. albendazole group vs. dexamethasone group vs. co-therapy group, * *p* < 0.05, ** *p* < 0.01) (BALB/c mice vs. C57BL/6 mice, # *p* < 0.05, ## *p* < 0.01, ### *p* < 0.001).

For meninges congestion and hemorrhage, BALB/c mice were found to have significantly higher scorings in received treatments from day 7 for 7 days (Figure 10a) (albendazole *p* < 0.05, dexamethasone *p* < 0.05, and co-therapy *p* < 0.01), those treated with albendazole (*p* < 0.001) and dexamethasone (*p* < 0.001) from day 7 for 14 days (Figure 10b), those treated from day 14 for 7 days (Figure 10c) (albendazole *p* < 0.001, dexamethasone *p* < 0.05, and co-therapy *p* < 0.001), and those with dexamethasone (*p* < 0.001). The co-therapy group of this murine strain had significantly lower scoring than the albendazole (*p* < 0.05) and dexamethasone (*p* < 0.01) group treated from day 7 for 14 days (Figure 10b) and had significantly lower scoring than dexamethasone (*p* < 0.01) group from day 14 for 14 days (Figure 10d).

**Figure 11:**
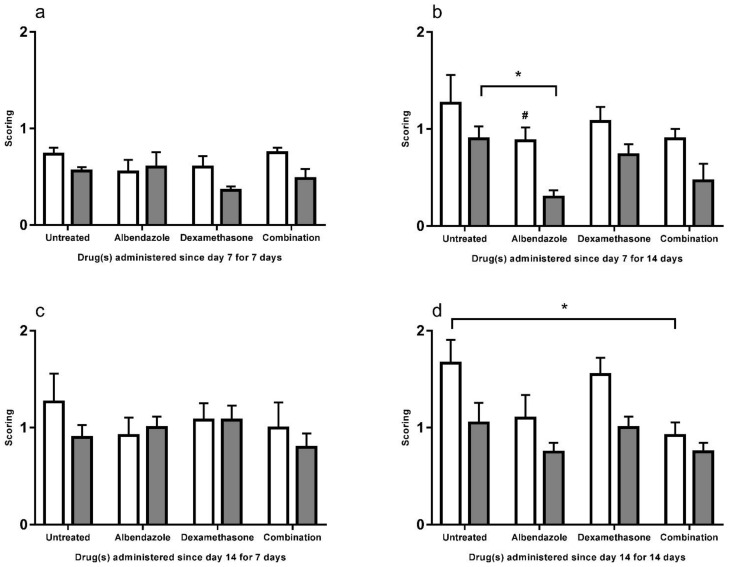
Scorings of size of larvae in the cerebral parenchyma of the BALB/c (white) and C57BL/6 (gray) untreated mice and those treated with albendazole alone dexamethasone alone, and co-therapy of the two drugs after infection with *Angiostrongylus cantonensis*. Data are expressed as mean ± standard error of the mean (Untreated group vs. albendazole group vs. dexamethasone group vs. co-therapy group, * *p* < 0.05) (BALB/c mice vs. C57BL/6 mice, # *p* < 0.05).

Although mice treated with dexamethasone were observed to have larger worm size (K of Figure 1, Figure 2, Figure 3, Figure 4, Figure 5, Figure 6, Figure 7 and Figure 8), no significant differences were found in the finding of larvae between the two strains of mice and among the treatment groups. However, the worms observed in the BALB/c mice were usually large in size. From day 7 for 14 days (Figure 11b), C57BL/6 mice treated with albendazole had significantly lower scoring than BALB/c mice (*p* < 0.05), indicating this drug has strong larvae removal effect in C57BL/6 mice. BALB/c mice had significantly lower scoring in co-therapy group than untreated group (*p* < 0.05) from day 14 for 14 days (Figure 11d).

### 3.3. RNA-seq

**Figure 12:**
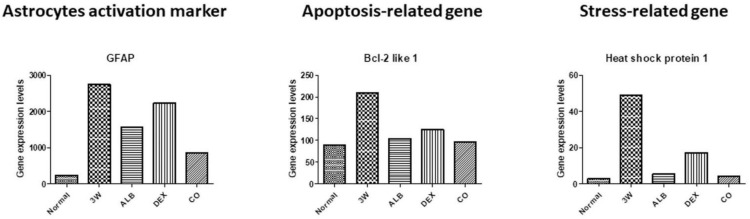
RNA-seq data of astrocytes activation marker, apoptosis-related gene, and stress-related genes after treatment of albendazole alone dexamethasone alone, and co-therapy of the two drugs to BALB/c mice infected with *Angiostrongylus cantonensis*.

**Figure 13:**
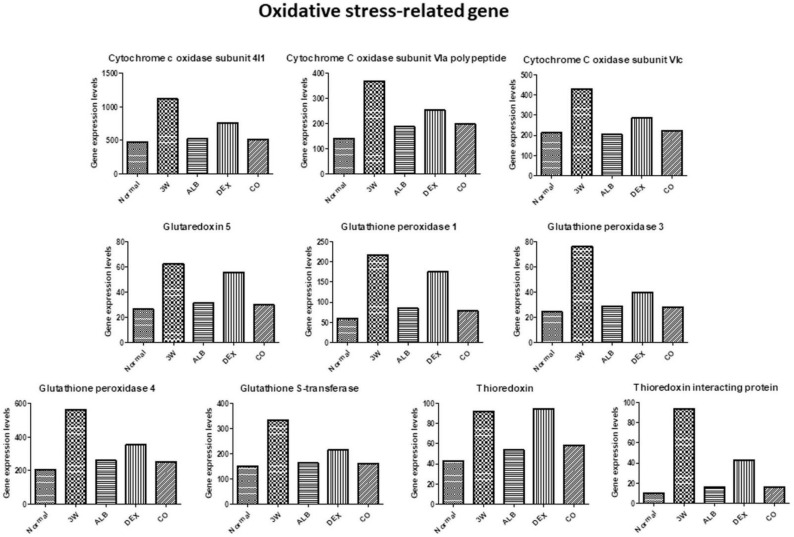
RNA-seq data of oxidative stress-related genes after treatment of albendazole alone dexamethasone alone, and co-therapy of the two drugs to BALB/c mice infected with *Angiostrongylus cantonensis*.

**Figure 14:**
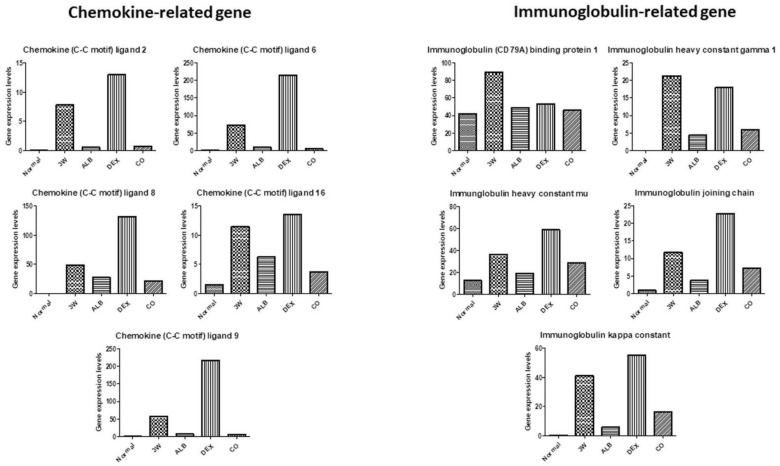
RNA-seq data of chemokine-related and immunoglobulin-related genes after treatment of albendazole alone dexamethasone alone, and co-therapy of the two drugs to BALB/c mice infected with *Angiostrongylus cantonensis*.

**Figure 15:**
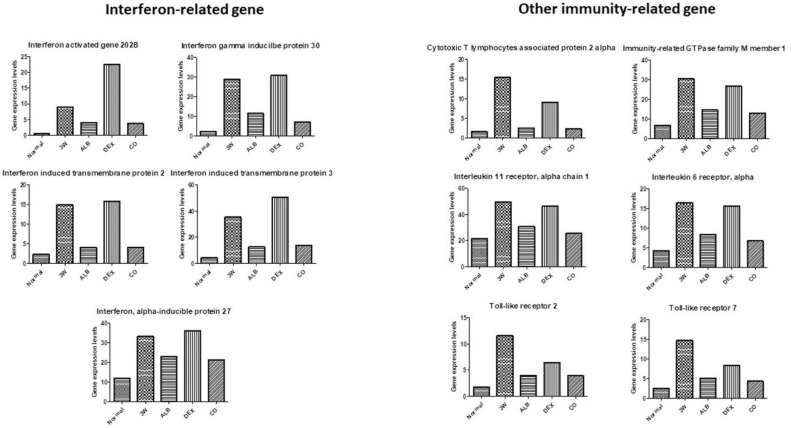
RNA-seq data of interferon-related and other immunity-related genes after treatment of albendazole alone dexamethasone alone, and co-therapy of the two drugs to BALB/c mice infected with *Angiostrongylus cantonensis*.

**Figure 16:**
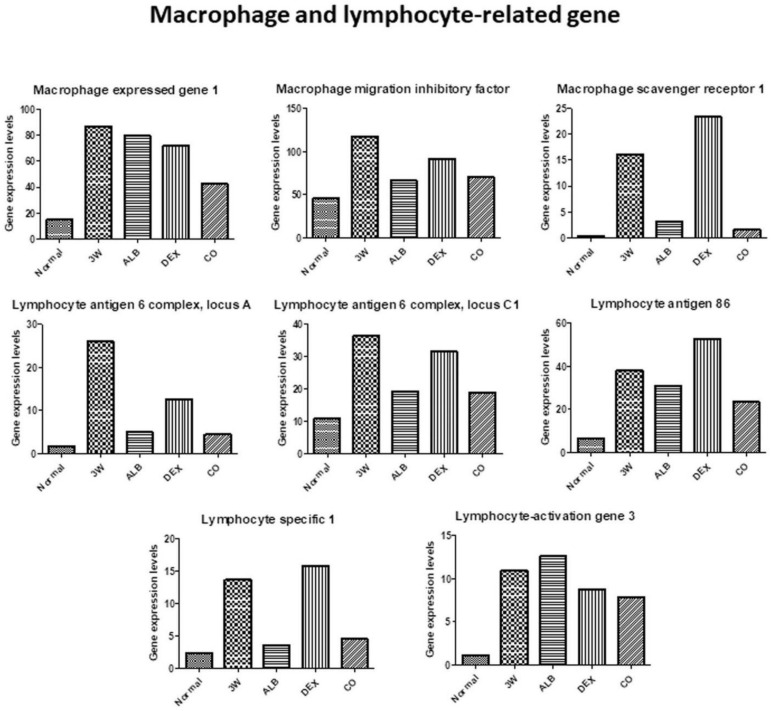
RNA-seq data of macrophage and lymphocyte-related genes after treatment of albendazole alone dexamethasone alone, and co-therapy of the two drugs to BALB/c mice infected with *Angiostrongylus cantonensis*.

Figure 12, Figure 13, Figure 14, Figure 15 and Figure 16 show the RNA-seq data. The expression levels of the astrocytes activation marker (glial fibrillary acidic protein), apoptosis-related gene (Bcl2 like 1), stress-related gene (heat-shock protein 1) decreased after treatment. The level in the co-therapy group was the lowest, followed by the albendazole group, and then by the dexamethasone group (Figure 12). Except thioredoxin, the expression levels of oxidative stress-related genes (cytochrome c oxidase subunit 4I1, cytochrome c oxidase subunit VIa polypeptide, cytochrome c oxidase subunit VIc, glutaredoxin 5, glutathione peroxidase 1, glutathione peroxidase 3, glutathione peroxidase 4, glutathione S-transferase, thioredoxin, and thioredoxin interacting protein) decreased after treatment and the levels in the albendazole group was similar to the co-therapy.

After dexamethasone treatment, levels of thioredoxin (Figure 13), chemokine-related ((chemokine (C-C motif) ligand 2, 6, 8, 9, and 16), some immunoglobulin-related (immunoglobulin (CD79A) binding protein 1, immunoglobulin heavy constant gamma 1, immunglobulin heavy constant mu, immunoglobulin joining chain, and immunoglobulin kappa constant) (Figure 14), interferon-related (interferon activated gene 202B, interferon gamma inducible protein 30, Interferon induced transmembrane protein 2, interferon induced transmembrane protein 3, and interferon, alpha-inducible protein 27), interleukin-related, toll-like receptor-related (Figure 15), and macrophage and lymphocyte-related genes (macrophage expressed gene 1, macrophage migration inhibitory factor, macrophage scavenger receptor 1, lymphocyte antigen 6 complex, locus A, lymphocyte antigen 6 complex, locus C1, lymphocyte antigen 86, lymphocyte specific 1, and lymphocyte-activation gene 3) (Figure 16) increased. The levels of these groups of genes decreased after albendazole treatment or co-therapy and the levels in the co-therapy group was the lowest in general (Figure 14, Figure 15 and Figure 16).

## 4. Discussion

*A. cantonensis* is an important parasitic zoonosis and cause damages in the central nervous system. Autopsies of patients with heavy infections have documented severe changes in central nervous system [31,32,33,34,35,36]. In the present studies, we have found eight important pathological changes in experimental infected BALB/c and C57BL/6 mice. Using the areas of these lesions, we are able to determine the severity of the infection quantitatively. Similar designs have been employed to study the pathological changes in the brain, cerebellum, lung, and pancreas of mice infected with *Neospora caninum* [37] and brain tissue changes in prolonged neonatal hypoxic ischemic brain disease [38]. The application of a scoring system enabled us to determine that drug administration strategy, time of drug administration, and number of days administered are important factors for the success of therapeutic measures against *A. cantonensis* infection. Moreover, the significant differences in the Th-1 and Th-2 dominant murine strains in the response of the treatment also indicate the co-therapy of an anthelmintic and a corticosteroid should be more effective and safer than using the drugs alone.

It has been reported that hemorrhage, immune cell infiltration, and congestion occurs more significantly in infected mice than in rats with *A. cantonensis* in weeks 3 and 4 post-infection [39]. After dead worms are surrounded by immune cells, granuloma and spindle-shaped Charcot-Leyden crystals were formed, suggesting the migration of the young adults of *A*. *cantonensis* to the host brain [4,40]. In this study, we also found the young adults in the cerebral parenchyma. Although immune cell infiltration or inflammation was found to be reduced in the dexamethasone alone group, worms revealed in the cerebral parenchyma were found to have a larger size. This phenomenon was not observed in the remaining two groups (Figure 11). Treating mice with steroids can effectively suppress the immunity, thereby reducing the impact on the worms and making the worms larger in size.

Among anthelminthics, levamisole [41], tribendimidine [42], mebendazole [43], and flubendazole [43,44] have also been evaluated to be effective against *A. cantonensis* in mice infected in the early stage. Moreover, albendazole is not only effective against *A. cantonesis* infection in mice but also has significantly higher intestinal absorption rates [5,6]. The action of this widely used anthelmintic is to interfere with glucose uptake of the parasite through the binding to β-tubulin and inhibiting its synthesis [45]. Albendazole enters the subarachnoid space to achieve the nematocidal effects [46]. In addition, albendazole is able to inhibit the activity of MMP-9, a factor causing damages of brain blood-brain barrier, immune cell infiltration and inflammation-related cytokine release, in the infected mice with *A. cantonensis* [9,10,12,13,47,48].

As an effective measure to reduce the inflammatory responses, steroids have been administered to patients with eosinophilic meningitis and eosinophilic meningoencephalitis. These drugs not only inhibit the inflammation-related transcriptional genes such as IL-3, IL-4, GM-CSF and the related chemokines but also reducing the survival of eosinophils [49]. Administration of prednisolone for two weeks may alleviate headache [50] and a one-week course of adrenocortical steroid may reduce inflammation [51]. Treatment of dexamethasone for 7 days may inhibit apoptotic proteins in the brain and prolong the survival of the mice infected with *A. cantonensis* [52,53]. In this study, we found that the results of administration of dexamethasone alone to the infected mice from day 7 were satisfactory (Figure 9a). However, the worms in the groups treated from day 14 were nearly intact with less meningeal infiltration and perivascular cuffing (Figure 3, Figure 4, Figure 7, and Figure 8). These findings indicate that dexamethasone may not inhibit the immune responses in the later stage of infection. Recently, spleen atrophy has been found in mice infected with *A*. *cantonensis* [52] and the infection may promote endogenous corticosteroid activity in mice [53]. These changes may cause immunosuppression in the host. Secondary infections of multi-drug resistant *Staphylococcus aureus* and *Clostridium difficile* have been reported in a severely ill patient with eosinophilic meningitis caused by *A. cantonensis* and treated with prednisone for a long period of time [54]. Dexamethasone may significantly reduce the immune functions of the host but not eliminate the parasite.

Co-therapy of albendazole with dexamethasone may increase plasma levels of albendazole by two folds, resulting in elevating the therapeutic effect of the anthelminth [55]. This therapeutic measure has been reported to reduce clinical manifestations. The expression of IL-5 mRNA in the peripheral blood has a decreasing trend and eosinophils returned to the normal level. The shifting of Th2 immune response to Th1 immune response was another therapeutic effect [12,13]. In addition, significant reductions in the levels of IL-5, TNF-α, IL-1β, and MMP-9 have been reported in infected mice treated with albendazole and thalidomide [8]. Combined treatment with albendazole and baicalin not only prolonged the survival rate but also elevated the weight of infected mice to the normal level [9]. In the present study, the size of worms and inflammatory responses significantly reduced in the co-therapy group. However, infected rabbits with albendazole alone may provoke severe pathological changes in the brain [7]. These findings indicate that co-therapy of albendazole and dexamethasone should be a more effective and safe alternative for the treatment of cerebral angiostrongyliasis.

BALB/c and C57BL/6 mice were well-known to have different morbidities and mortalities for *A. cantonensis* infection. BALB/c mice were susceptible to the infection whereas C57BL/6 mice were relatively resistant [56]. Comparing these two murine strains, Th2 cytokine responses, especially those involving IL-5, were determined to be more predominant than Th1 cytokine responses in the infected mice. However, the association between host morbidity or mortality and cytokine responses could not be clearly defined [57]. Nevertheless, the morbidity of infected BALB/c mice was found to be regulated by some unknown CD4+ T-cell-dependent mechanisms and IL-5-, eosinophil-, or TNF-alpha-dependent mechanisms could be ruled out [58]. Recently, we employed the immunohistochemical technique to determine the temporal-spatial expressions of IL-4, IL-10, and IL-13 in the brains of infected BALB/c and C57BL/6 mice. The special temporal-spatial expression changes of these three cytokines in the cerebral parenchyma provided explanation the differences in the survival and the time of occurrence of immune responses in the infected hosts [15]. In this study, we also revealed that C57BL/6 mice had significant pathological scoring than the BALB/c mice and the therapeutic effects of the three drug administration strategies were also higher in the former than the latter. These findings suggest that there should be no significant differences in the actions of the administration strategies against *A. cantonensis* between Th-1 and Th-2 dominant mice.

RNA-Seq is a technique based on the next-generation sequencing in high-throughput analysis of genes at the transcriptomic level. It had been widely used in the treatment and diagnosis of many diseases, especially cancers and genetic-related diseases [59]. Among parasitic infections, it has been successively applied to analyze the spread of malaria [60] and detect *Plasmodium* and *Leishmania* species with drug-resistant at an early stage [61]. In the present study, we used this technique to detect the immune responses in *A. cantonensis* infected mice before and after the administration of the three therapeutic strategies at the gene level.

By RNA-seq, we can evaluate the effects of the therapeutic strategies at the transcriptome level. In general, the levels of these genes decreased after albendazole treatment or co-therapy and the latter were lower than the former. The levels in the mice treated with dexamethasone alone remained high or even higher than those in the untreated group. Based on the findings in the present study, co-therapy of albendazole and dexamethasone should be an effective and safe alternative for the treatment against *A. cantonensis* infection.

Up-regulation of GFAP is an important character of the active inflammatory state of the astrocytes [62]. After *A. cantonensis* infection, the expression of GFAP in the brain of infected mice increased, indicating that the infection is able to cause damages to the central nervous system and activate astrocytes for repairing and regeneration. Among the treated mice, those treated with dexamethasone alone had a higher GFAP expression level, suggesting that administration of steroids alone may not have beneficial effects to recovery of brain damages (Figure 12). In addition, mice infected with *A. cantonensis* showed that Sonic Hedgehog activates Bcl-2 through the GRP78-dependent pathway to enhance the activities of astrocytes and inhibits cell death [26]. The co-therapy may have better therapeutic effects, since the expression of the apoptosis-related factor Bcl-2 like 1 and the cellular stress factor Hsp 1 of mice in this group reduced to the lowest level (Figure 12).

Increase in the oxidative stress has been reported in mice infected with *A*. *cantonensis*. The production of reactive oxygen species (ROS) may cause brain damages associated with meningitis [63]. By RNA-seq analysis, we revealed that the expression of oxidative stress-related factors in the brain of the infected mice increased. Moreover, no reduction in the expressions of these factors was found in the dexamethasone group (Figure 13). Brain damages may be caused by the production of ROS and cytokines, which in turn activate the MEKK1/JNK and JAK/STAT1 pathways. In addition, NF-κB and JNK may be activated by induction of IAP. NF-κB binding protein translocates to the nucleus and activates cytokine. These changes lead to activation of the JAK/STAT pathway. The activation of JNK is triggered by proliferation and inflammation in the brain tissue. The signal transmission pathways of JAK/STAT1, IAP/NF-κB, IAP/JNK and MEKK1/JNK may play an important role in initiating and/or enhancing the brain injury associated with *A. cantonensis* infection [47]. Cys-Cys (CC) type chemokines, such as CCL2, CCL8, CCL1, CCL24, CCL11, CCL7, CCL12, and CCL5 had been demonstrated to increase significantly. The increase in CCL2 indicates that the worms have already migrated to the brain in the early stage of infection and causes neuropathic pain. At the late stage, the increased expressions of IL-4, IL-5, IL-10 and IL-13 are immunopathological responses mainly regulated by the Th2 immune responses. The cytokines may form a network-like function of mutual regulation and inhibition and cause eosinophilic inflammation [64]. By RNA-seq, immune-related factors, such as chemokines, immunoglobulins, interferons, and cytokines, were revealed in infected mice. The co-therapy groups were found to have the highest reductions in the expressions of these factors (Figure 14 and Figure 15).

The infected mice had dramatically increased in the level of Chi3l3 in the course of *A*. *cantonensis* infection. The inflammatory macrophages secreting Chi3l3 increased proportionally to eosinophils in the brain. Moreover, the soluble antigen of *A. cantonensis* may induce brain eosinophilic meningitis through aggravating a positive feedback loop between IL-13 and Chi3l3 [65]. In this study, macrophage and lymphocyte-related factors increased after infection. However, levels of other immunity-related factors in the dexamethasone group remained high and only MPEG1 and LAG3 remained high in the albendazole group (Figure 16).

In the corticosteroid group, the inflammatory responses in the brain were significantly suppressed. However, severe bleeding occurred, and the larvae were found to have the largest size. Although the level of immune responses reduced, the apoptosis, oxidative stress, and other immunity-related factors remained at high level. Moreover, no reduction in GFAP was found after treatment, indicating that no improvement in the damages of the central nervous system. Co-therapy showed the highest effectiveness by histopathological and RNA-seq analyses. It is able to simultaneously eliminate the worms and inhibit the inflammatory responses.

## 5. Conclusions

Based on the results of the present study, co-therapy of albendazole with dexamethasone is more effective than administration of albendazole or dexamethasone alone against *A. cantonensis* infection. Moreover, the co-therapy strategy significantly reduces pathological changes post-infection.

## Data Availability

All data generated or analyzed during this study are available from the corresponding author upon reasonable request.
